# Do women and men compete equally on a level playing field? An empirical investigation into the 2021 Olympic shooting competitions

**DOI:** 10.1371/journal.pone.0291017

**Published:** 2023-09-27

**Authors:** Nadav Goldschmied, Abraham García-Aliaga, Diego Muriarte Solana, Daniel Mon-López

**Affiliations:** 1 Department of Psychological Sciences, College of Arts and Sciences, University of San Diego, San Diego, California, United States of America; 2 Departamento de Deportes de la Facultad de Ciencias de la Actividad Física y del Deporte-INEF, Universidad Politécnica de Madrid, Madrid, Spain; University of Alabama in Huntsville, UNITED STATES

## Abstract

Due to physical differences between the genders, it is hard to study whether women also vary from men in their competitive drive in sports. The Olympic committee instituted major rule changes in the sport of shooting in the Tokyo Olympic Games (2021), leveling the playing field. We explored performance in a myriad of competitions including newly established mixed-gender doubles events in this unique sport of very limited physical input. Men performed better than women in events which required “dynamic” qualities (following moving targets) but when the competitions were held most constant (rifle shooting with stationary targets indoors) and regardless of distance or posture, women performed equally to men exhibiting seemingly similar competitive drive. The last finding should propel the Olympic committee to fully integrate rifle events. In the broader sense, we find that when the playing field is even, the genders, at least among highly trained selective competitors compete equally.

## Introduction

Sometime in the early 2000’s Uri Gneezy and Aldo Rustichini conducted a very intriguing field study in an elementary school in Israel [1]. The participants were prepuberty140 children, 75 boys and 65 girls, all in the fourth grade between 9–10 years of age. The researchers studied the performance of the children in a race alone over a short distance of 40 meters (~131 feet) with the teacher measuring their speed. Girls and boys ran on average at the same speed. Then the majority of the children ran a second time with the teacher matching the children in pairs, starting with the two fastest children in the race going down the list independent of gender. Each pair ran on the same track, with the two children running alongside this time. Now, the boys improved while the girls ran slower. In eight mixed-pair races of 11 observations (73%) in which boys were slower than the girls initially, they beat the competition in the head-on second stage. In the remaining 18 mixed-pair races, where the girls had a worse time in the first round, only three girls won the competition (17%). To combat experimental threats, the researchers wisely kept a separate group of children as controls who ran alone in round two as well. This group, yet again showed no gender differences in speed and thus dispelled alternative explanations such as girls getting tired faster than boys. Based on the results, Gneezy and Rustichini concluded “Overall, we find support for the claim that competition increases the performance of males relative to females…This indicates that some strong, robust, and general factors are involved.” They then raised further: “The puzzle that remains concerns the more subtle effects of competition in homogeneous and heterogeneous groups.” (p. 380).

Notwithstanding experimental concerns in regards to the criterion of who was assigned to the control vs. the experimental condition, time measurement [2], possible disparate crowd response in competition based on gender [3] and failure to replicate [4,5], gender differences in sport competition are of paramount interest (the Gneezy and Rustichini’s study was cited more than a 1000 times) and are worth studying to explicate whether they are propelled by inherent differences or are the product of social/ environmental circumstances. In the current exploration, we utilize a highly controlled environment with homogeneous groups to shed more light on this old age question.

Abundant of research in this field is still in agreement with the results of the original running study, arguing that when the stakes are especially high males show greater competitive tendencies, which lead them to superior performance over females. Three main categories of research make this claim. Laboratory experiments [6,7], archival data [8,9], and professional sport performance research [10,11]. Since the focus of the current investigation falls within the latter research purview, we next outline research of gender comparisons in sports. It is, of course, important to note that men after puberty have stronger physical attributes (e.g., muscle mass, height, aerobic and anaerobic capacities, and hemoglobin levels) which accord them great advantage in top tier sport performance [12–14]. These differences, sensibly, keep the genders apart in almost all elite sport competitions and make it harder to assess if there are also innate gender differences in their competitive drive.

### Gender differences in sport performance among elite athletes

Due to the limiting circumstances of gender separation, which preclude direct comparisons, researchers need to show ingenuity in exploring gender differences in sport. Also of note is the type of sports studied whereupon some competitions are characterized as closed skill (i.e., occur in fixed or predictable situations) [15,16] such as running, swimming or rifle shooting while others are more open and interactive (i.e., affected by the opponent’s maneuvering). This distinction (or more precisely a continuum of skills) may interact with gender to produced disparate outcomes.

In tennis, an interactive sport, Paserman [11] studied nine Grand Slam tournaments (2005–2007) and found that both genders declined in performance in the final and decisive set (controlling for the length of the match). However, in a subset of these games a point-by-point analysis showed that women were more likely to commit unforced errors when the point was of greater importance, while men’s were unaffected. Paserman argued that women adopt a too cautious and less aggressive strategy when the stakes are especially high. More recently, in a study of the 2012 ATP and WTA tennis tours [17] found that women were not more likely than men to lose in straight sets but those who did, won fewer games in the second set than men did. It was also determined that women who lost the second set after winning the first one were more likely than men to withdraw from the third set. Overall, the researchers concluded was that women were not more likely to lose because of setbacks and not more easily discouraged than men. Another tennis study conducted a comprehensive analysis of 3,844 men’s sets and 3,034 women’s sets [18], and found that women’s sets were less competitive (tight) than men’s with a lower average number of games in a single set. However, when controlling for height and body mass index (BMI), the gender difference in the tightness of the final score disappeared and the researchers concluded that the gender difference in the final score of tennis sets arose from gender differences in physical power and not competitiveness.

Shifting to more closed and continuous type of sport, performance in New York City Marathon (2007–2014) was studied where female runners start the race 30 minutes earlier than their male runners, which leads some elite women to be eventually passed by the fastest men [10]. It was found that as the men overtook the female runners, the performance of the surpassed female runners declined 1.9% relative to the mean based on 5km splits analysis. However, the decline was different across ability level, with the largest drops in speed among lower ability runners. It is also worth to emphasize that the best female runners were never overtaken by men and thus excluded from this analysis.

Other research in gender differences in professional sports focused on financial rewards and their impact on performance [19–23]. For example, in the Ladies Professional Golf Association (LGPA), a sport which is characterized by closed and internally paced skills (i.e., the initiation of the performance is greatly determined by the performer), it was found that an increase of the total prize money led to a weaker performance as measured by an increase in the number of attempts to complete the course. This trend possibly indicates women yielding to the pressure that comes with large prizes at stake. Other research found that men professional golfers (PGA) improved their performance when financial rewards were higher [24,25]. However, a more recent study failed to replicate the men’s results with the 1992 PGA records [26] and the overall results in this domain of research are mixed.

Most closely approximating the sport of shooting, Duffy [27] in quasi-experimental work found that in dart throwing, a sport, which values precision over power, elite men performers consistently, outdid their female counterparts. This gender gap also correlated with archival data from real competitions. It is also worth noting that when Duffy accounted for height and arm length, there were still significant gender differences in dart throwing performance.

Specifically in rifle shooting, the focus of the current investigation, competitors perform in a highly controlled environment. The sport is characterized as closed in terms of skill execution where the environment is mostly predictable and static [28], with fine motor (e.g., trigger pull) and largely internally paced performance initiation as well as discrete execution (i.e., clear beginning and end as opposed to continuous). A longitudinal study compared the shooting performance of male and female competitors during the National Collegiate Athletic Association (NCAA) Rifle Championship from the 2007 to 2013 seasons [29]. NCAA competitions, distinct from Olympic shooting events at the time, bring male and female shooters to compete against each other. Utilizing archival investigation of both the team and individual tournaments, 555 scores of the best 149 shooters showed no differences in performance between the genders during team as well as the individual (best performers) events.

In the same sport, an important rule change in 2018 was examined when the Olympic shooting protocols increased the number of women’s shots from 40 to 60, making them equal to men [30]. This study which included 292 shooters contrasted in the 2016 (40 shots) and/or 2018 (60 shots) European Championships analyzing the top-50 results. Men and women shot equally well with rifles although the men’s performance with pistol was higher than that of women (the women’s performances did not decline for the pistol or the rifle category when their number of shots were increased in the 2016 to 2018 events). The researchers concluded “that sports in which physical strength is a minor factor, as in the case of shooting, should revise their regulations in the interest of greater gender equality in sports.” (p.1). However, there are some technical and physical skill differences between precision and clay target modalities that could be relevant in the performance and no previous studies have compared other shooting modalities.

In the current investigation, we endeavored to test this conclusion when the stakes are highest during the Olympics where contestants can win substantial sums of money [31] and sponsorship deals could be financially life altering as well as earn tremendous personal prestige, perhaps for the rest of their lives, from having beaten the best. Aside from leveling the playing field for the genders, the Tokyo games allowed for a comprehensive comparison going beyond only air-rifle [29] or air-rifle and pistol shooting [30] events. The additional competitions included skeet, trap and 50 meters 3 positions events that allowed for a more nuanced exploration of gender differences in performance since the first two incorporate a “dynamic” component (i.e., following and hitting moving targets) while the latter requires diversity of shooting postures and distance (in contrast to other events). Lastly, the recent Tokyo Olympics introduced an influx of “mixed-double” team events whereupon two gender duos were vying for the gold. These events reflect a major push towards equality of participation and opportunity in this sport with similar rate of participation of men and women shooters.

In the way of specific directional hypotheses, we predicted based on past research [29,30] that the genders would perform equally in the air-rifle competitions but not in the pistol shooting event. We extrapolated similarity in gender performance to the 50 meters 3 positions event due to its static nature. In the absence of a direct comparison to the “mixed-double” events we regarded our hypotheses as exploratory. On the one hand, there is sufficient research to anticipate women performing worse under co-acting circumstances when accountability his high [10,19,20] but also contradictory empirical evidence. For example, in biathlon women exhibited shorter (i.e., better) shooting times in the presence of an audience than in the absence of audience, whereas men showed an opposite trend [32].

## Methods

### Participants

This study was approved by the Ethics Committee of the Polytechnical University of Madrid. The participants of the study were 357 shooters of 101 countries. Of the total participants’ number, two shooters were excluded because one was disqualified, and the other did not start the competition. The final sample was composed of 355 shooters with an average age of 29.9 ±7.65 years (Min = 17, Max = 64). The gender distribution was 178 women and 177 men.

The qualification system for the mixed team events according to the ISSF rules is as follows: “Mixed Team entries may come from athletes entered in Mixed Team quota places obtained at the 2018 WCH Changwon, KOR and from double starters who are also entered in individual events.” (ISSF, 2020). In consequence, those athletes qualified in the mixed events during the world Championship in 2018 were directly classified for the Olympics in the mixed events. In addition, those shooters who qualified in the individual’s events with an opposite gender mate who qualified independently in the same event, were able to join forces in the mixed event. For example, a male air pistol shooter and a female air pistol shooter who has qualified in the individual event could participate in the mixed event together. Lastly, it is important to note that the individual events were finished before the mixed events had commenced as to not undermine performance in the individual modalities. Shooters who qualified for the team competition hence increased their chances of winning a medal without jeopardizing their focal individual pursuit.

**Fig 1:**
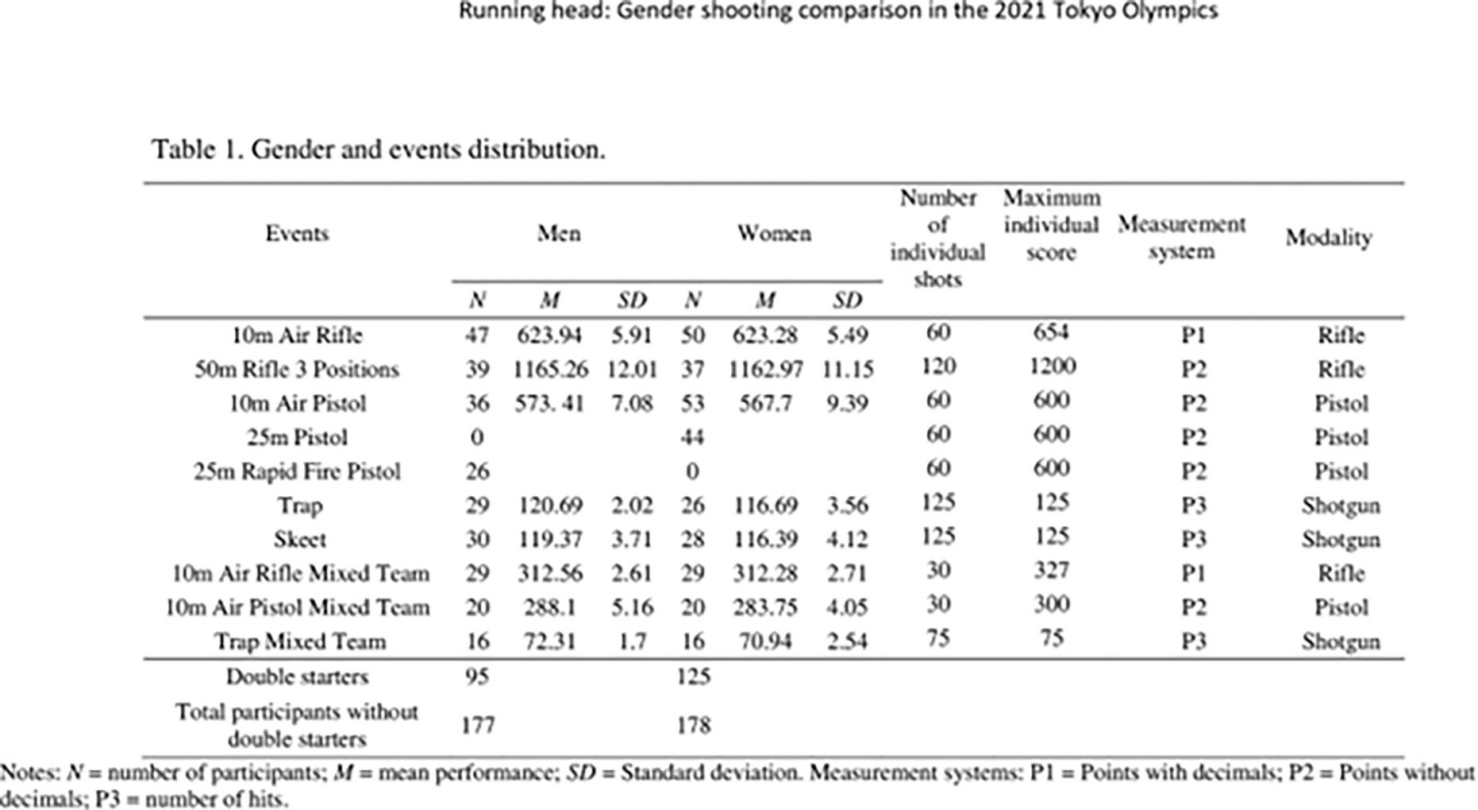
Gender and events distribution.

The participants took part in a total of 15 shooting modalities, five modalities for each weapon: pistol, rifle, and shotgun. Additionally, six shooting modalities were for men, six for women and three for mixed team modalities. Among the competitors, 95 men and 125 women were double starters, competing in two events. **See Fig 1.**

### Procedure

The data of the present study were collected during the Olympic Games Tokyo 2021 from July 22^nd^ to August 2^nd^. The results were tabulated from the International Shooting Sport Federation (ISSF) webpage once the results became official (Federation, 2021).

According to the new International Olympic Committee competition protocol, the number of shots allotted were matched and to balance the competition by gender. In addition, prone rifle, free pistol and double trap modalities were replaced by three mixed team modalities in the Tokyo 2021 schedule (air pistol, air rifle and trap mixed team), in which both men and women competed together and simultaneously [30].

As inclusion criteria, only those modalities in which men and women fired the same number of shots and under the same conditions, both individually and in mixed teams, were selected to be analyzed. Consequently, the modalities of rapid-fire pistol (only men) and sport pistol (only women) were excluded from the study.

Additionally, the final rounds were excluded from the analysis due to the specificity of these stages’ rules and the low number of participants (from six to eight depending on the modality), as they could entail insufficient statistical power.

Regarding the qualification round structure, athletes fired 60 shots in sets of 10 shots for individual events of air pistol and air rifle (both men and women) while mixed events of air pistol and air rifle have a total 60 shots per team in sets of 10 shots, (each gender shot 30 shots). Additionally, 3x40 rifle event consist in 120 shots in sets of 10 shots divided 40 shots by position (prone, knee and standing). On the other hand, clay target individual modalities of trap and skeet consist of 125 targets in sets of 25 shots (both men and women) while, in the mixed trap team event, each gender shot 75 targets for maximum score of 150 hits. See Fig 1.

### Variables

Performance were measured using the total points in the precision events and total number of hits in trap and skeet events according to the ISSF rules and regulations (ISSF, 2020). Furthermore, to make possible the performance comparison between modalities with the same weapon but with different number of shots (for example: air pistol vs. air pistol mixed team and air rifle vs. air rifle mixed team) the average points per shot was calculated [30].

Performance outcomes were measured using the total points (continuous score) in the precision events. More specifically, points with decimals in 10m Air Rifle and 10m Air Rifle Mixed Team and points without decimals in the rest of events. The total number of hits (a fraction score) was computed for the trap and skeet events and trap mixed team events according to the ISSF rules and regulations (ISSF, 2020) [see Fig 1].

### Statistical analysis

The data are described by arithmetic mean (*M*) and standard deviation (*SD*). The normal distribution of the variables was checked using the Kolmogorov–Smirnov and Shapiro–Wilk tests.

**Fig 2:**
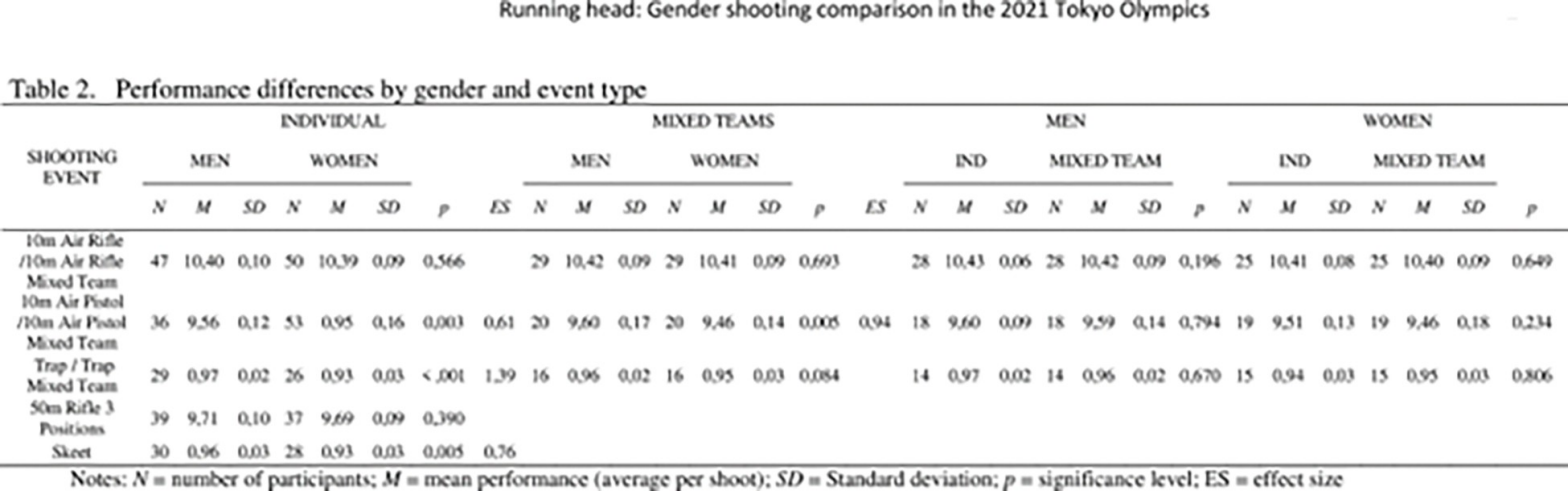
Performance differences by gender and event type.

For descriptive purposes, the cells means by gender and type of competition are presented in Fig 2 (as well as the *T* tests used to contrast gender and competition type). In our main analysis, we utilized A two-way mixed ANOVA to analyze the differences between gender (male vs. female), the shooting event (individual vs. mixed team), and the interaction between them. Effect size with a 95% interval confidence was calculated using Cohen’s *d* and partial eta squared. Three benchmark points were defined to indicate the effect size, (*d* = 0.2 small, *d* = 0.5 medium, *d* = 0.8 large) and partial η2 = 0.01 small; η2 = 0.06 medium; η2 = 0.14 large. (Cohen’s, 1988). All statistical analysis was conducted with SPSS version 28.

## Results

### Comparison by gender and event competitions

The comparisons between gender in the individual competitions showed that men shot better than women in 10m air pistol *(t*_(87)_ = -3.1; *p* = 003; *d* = 0.60, IC95% (0.52–0,70)); skeet (*t*_(56)_ = -2.89; *p* = 005; *d* = 0.76, IC95% (0.23–1.30)); trap *(t*_(38.61)_ = -5.04; *p* < 001; *d* = -1.38, IC95% (0.80–1.98)). Contrary, no differences by gender were found in 10m air rifle nor 50m rifle 3 positions (*p* > .05). In order to increase confidence in the overall reliability of the results we also conducted a repeated bootstrapping procedure. The results remained the same for the t-tests with a simulation of 2000 samples and a 95% confidence interval.

In addition, the comparisons between gender in the mixed competitions showed that men perform better than women in10m air pistol (*t*_(38)_ = -2.97; *p* = .005; *d* = 0.94 IC95% (0.29–1.59). No differences by gender were found in trap nor 10m air rifle (*p* > .05). Also, no differences were found between the individual and mixed competitions in any event by gender (*p* > 0.05). **See Fig 2.**

### Combined analysis

We further wanted to compare gender performance between the individual and the mixed events and the possible interaction between gender and events, which have different number of shots using the average points per shot. Consequently, a 2X2 mixed ANOVA analysis of variance with gender as the between-subject variable and type of competition (individual vs. mixed) as the repeated measure was carried out. See Fig 2.

For the 10-meters pistol shooting events (men = 18, women = 19) gender has a main effect (*F*_(1, 35)_ = 8.33; *p* = .007; partial η2 = .19) with men (*M* = 9.59) performing better than women (*M* = 9.48). There was no main effect of type of event on shooting performance (*F*_(1, 35)_ = 1.32; *p* = .260; partial η2 = .04), nor was there an interaction (*F*_(1, 35)_ = .73; *p* = .400; partial η2 = .02), showing that women did not deteriorate further in the mixed events beyond their individual performance level.

In the trap shooting events (men = 14, women = 15) a significant gender effect was found (*F*_(1, 27)_ = 6.29; *p* = .018; partial η2 = .19) with men (*M* = 0.96) performing marginally better than women (*M* = 0.94). There was no main effect of type of event on shooting performance (*F*_(1, 27)_ = .007, *p* = .940, partial η2 = .00), nor was there an interaction (*F*_(1, 27)_ = .21; *p* = .65; partial η2 = .008), showing that women did not deteriorate further in the mixed events beyond their individual performance level.

Regarding the 10-meters air-rifle shooting events (men = 28, women = 25) our results showed no main effect for gender (*F*_(1, 51)_ = 1.06; *p* = .308; partial η2 = .02). There was no main effect of type of event on shooting performance (*F*_(1, 51)_ = 1.36; *p* = .250; partial η2 = .03), nor was there an interaction (*F*_(1, 51)_ = .17; *p* = .680; partial η2 = .003), showing that women did not deteriorate in the mixed events in comparison to their individual performance level.

## Discussion

We found superior performance for men in most shooting events except in the rifle modalities (10 meters air rifle and 50 meters rifle 3 positions events). The latter competitions are unique as they afford the most control (initiation of trigger pull and stationary targets indoors) as well as physical support (the rifle is held close to the trunk of the body and a support vest is sustaining the weight of the rifle). These findings lead us to the conclusion that when movement is kept minimal and regardless of distance or posture, women are not less competitive than men are, and they perform equally. This was true both when they compete in gender segregated events as well as in the mixed-team competition. It is important to note that by no means do we deem rifle events to be “easier” or less demanding in comparison to other shooting events. Rifle elite shooting requires intense focus and sustained effort [33] as well as superior technical-coordinative skills [34]. An exception to this distinction between “still” vs. “dynamic” shooting in gender performance is pistol shooting whereupon while still being static in nature, males perform better than females. This finding is line with past research in dart throwing [27] where, similarly, a discrete skill is executed further away from the trunk of the body.

However, the results should be also analyzed through a much broader lens extending beyond Olympic shooting competitions. They show that under unique and probably not easy to attain set of circumstances, highly trained experts of both genders compete equally. Still it seems that even minor barriers may cause uneven competitive environment. Coming full circle to Gneezy and Rustichini’s [1] contemplative musing about gender differences cited earlier, we find that when the sample is homogenous (i.e., elite shooters) and physical barriers are not present, we find no difference in competitive drive between the genders utilizing an indirect research design. Yet again, it is important to qualify our conclusions as the sport of shooting is a consistently ‘do you best’ type of competition which does not involve strategy or risk taking considerations. Past research [35,36] is mostly in support of gender differences in regards to these two elements (but see [37] for an exception).

A psychological analysis contrasting the “mixed-double” events with the individual competitions highlights several important dimensions [38]. In the former, performance is done in close physical proximity to one’s opposite gender teammate with an exact knowledge of score progression and thus is likely to generate team accountability pressure. While we usually observe better overall shooting performance by men (except for the rifle competitions), women in the “mixed” events did not decline in their team performance. Their accountability towards a usually better shooting male teammate, was not a liability and women performed on par with their individual records. On the other hand, the mixed-doubles competition may not be considered as direct gender competition by the shooters as each competitor may perceive to nullify his/ her contribution with the opposite gender competitor. This psychological perspective (if true) may alleviate stereotype bias concerns [39] which may arise otherwise.

We choose to pursue our gender exploration based on average performance analysis since this approach was most robust and valid statistically allowing maximum power to test the hypotheses under the current sample size limitations. However, Olympic medal winning competitions (and other sports) are a case of outlier performance [40]. In other words, the genders on average may perform equally but possibly none of the top performers qualifying (or winning medals) would be women. An inspection of the individual performers in Tokyo negates such an unlikely scenario as five among the top eight performers in the 10 meters rifle preliminary competitions were females (men and women’s scores combined). A similar scrutiny in the 50m rifle 3 positions competitions yielded four hypothetical women qualifiers–exactly half of the advancing field. Moreover even when male competitors are found to be better in skeet shooting historically and based on our current findings, in past games a Chinese shooter, Zhang Shan, overcame three male opponents in the finals to win the then the fully mixed-gender competition (before the genders were separated possibly because of her spectacular triumph).

Although this is the first study that have analyzed gender performance differences comprehensively in all shooting modalities, general limitations should be pointed out such as the Covid 19 pandemic timing which disrupted practice and competition routines [41] as well as the possible gender differential impact of the contagion [42]. In addition, we focused solely in our investigation on gender and did not include other variables, for example, the shooter’s country of origin. Furthermore, these two factors may interact, as observed in other research whereupon a country’s gender gap status served as a predictor for diverging sport performance [3]. Future research with more data should expand the exploration to paint a broader picture of elite performance when the stakes are high(est).

More specifically, it is important to note that the number of shooters who participated in both, the mixed and in the individual competition were smaller than those who competed only in the individual or the mixed competitions, a dimension that could reduce our statistical power. Nevertheless, the results of this study corroborate earlier findings from the world championship event [30] and collegiate competitions [29] and should lead the way, in our opinion, for the Olympic governing body to make the leap forward and turn the rifle-shooting events to fully integrated and equal competitions whereupon the genders compete directly against each other. Such policy change, naturally, bares also risks, as primarily it would objectively reduce medal opportunities for women (as well as, possibly, for men). It is important to note that gender segregated events were historically put in place to increase opportunities for women to compete in general. If a new direct competition policy is indeed enacted, it should subsequently be reevaluated to assess the impact of this monumental change, as our results are circumstantial (i.e., the genders did not compete one against the other in the individual events).

Two major consequences arise if indeed in a direct competition scenario, the genders perform equally. First, recent years highlighted the controversy around the participation of trans competitors in sports [43]. This contentious debate should become mute in rifle shooting saving the trouble of ascertaining male vs. female classifications and making a significant advancement into a sex-neutral era [44]. Second, female Olympic role models should emerge. A recent national survey in the USA found that a third of parents still believed that boys were better in sports and more competitive than girls [45]. Past research of role-models in other domains [46,47] demonstrated the potent force of these distinguished figures to inspire next generations of young girls to compete in the pursuit of excellence and in shattering socially constructed traditionally male-dominated fields.

## Conclusions

Women and men shooters performed separately but equally in the 2021 Tokyo Olympics in “static” rifle shooting modalities. Men were superior in “dynamic” (i.e., moving target) shooting events. In the newly formed “mixed” team events (one male and one female shooters competing alongside) these performance patterns were maintained and the mixed gender competitive environment did not impede women’s performance beyond. Supported by earlier research [29,30] we endorse the proposition that in future Games, “gender unified” events should be held for the “static” rifle shooting modalities.

## Supporting information

S1 File (SAV)
